# Closing the monitoring gap: validation of a novel micro‐electromechanical systems (MEMS) humidity‐sensing device for continuous respiratory monitoring during patient transfer

**DOI:** 10.1002/anr3.70057

**Published:** 2026-03-19

**Authors:** D. Rowe, M. Rowe, P. Melksham

**Affiliations:** ^1^ School of Medicine Griffith University Brisbane Australia; ^2^ Faculty of Medicine University of Queensland Brisbane Australia; ^3^ Department of Anaesthetics Wesley Private Hospital Brisbane Australia

**Keywords:** capnography, humidity, monitoring, patient transfer, respiratory rate

## Abstract

Transfer from the operating theatre to the post‐anaesthesia care unit is a high‐risk period with reduced respiratory monitoring, as conventional technologies are often impractical during patient movement. We evaluated a novel, low‐cost respiratory monitor using a micro‐electromechanical systems humidity sensor to detect breath‐by‐breath ventilation in spontaneously breathing adults. In this pilot validation study, 50 healthy volunteers wore a prototype humidity sensor mounted on a standard oxygen face mask delivering 4 l.min^−1^ supplemental oxygen, with the device generating a visual light signal for each detected breath. Respiratory rates measured over 60 s were compared with capnography, and agreement was assessed using Bland–Altman analysis and Pearson correlation. The prototype achieved a breath detection accuracy of 98.8%, with a mean bias of 0.16 breaths.min^−1^ and 95% limits of agreement from −1.17 to +1.49 breaths.min^−1^. Strong correlation with capnography was observed (r = 0.98, p < 0.001), and complete agreement occurred in 47 participants (94%), with discrepancies in three cases attributed to poor mask fit. This device provides an accurate, portable and inexpensive method for respiratory monitoring, offering continuous visual assurance when conventional capnography is unavailable or impractical during patient transfer.

## Introduction

The transition from the operating theatre to the post‐anaesthesia care unit (PACU) is a period of significant patient vulnerability. Despite the high acuity of such patients, the Seventh National Audit Project of the Royal College of Anaesthetists (NAP7) recently reported that respiratory monitoring was notably deficient during this time and can contribute to cardiac arrest [1]. This is a critical time for monitoring, as respiratory depression in this phase can be driven by residual neuromuscular blockade, opioids or volatile agents [2]. Instead, portable pulse oximetry is often used; however, this can result in late identification of compromise, in situations where early detection is vital [3].

There is often a reliance on manual respiratory rate assessment during patient transfer which is unreliable and frequently inaccurate [4]. Furthermore, although capnography is the reference standard, its use during transfer is limited by equipment bulk, set‐up time and power requirements [5]. In spontaneously breathing patients receiving supplemental oxygen, side‐stream CO_2_ sampling is also frequently degraded by gas dilution, which can mask hypoventilation or trigger false‐negative results [6].

Efforts to bridge this monitoring gap using impedance pneumography, acoustic sensors, wearable accelerometer‐based devices and many others have struggled with motion artefacts and complex set‐up requirements [7]. In the high‐paced theatre‐to‐PACU environment, any technology requiring calibration or external displays risks being bypassed in favour of visual observation [7, 8, 9]. This remains a significant safety concern given how frequently respiratory deterioration occurs, with some studies citing up to 30% in postoperative patients [10].

Since many postoperative patients already require supplemental oxygen, a potential monitoring solution lies in detecting the exhaled humidity which exits the lateral ventilation ports of the mask. Exhaled breath is nearly saturated with water vapour, creating a distinct cyclic moisture gradient relative to drier ambient air or medical oxygen [11].

Recent advances in micro‐electromechanical systems (MEMS) have enabled a new generation of highly sensitive sensors with ultra‐low power consumption and response times in milliseconds [12, 13]. We theorised that this technology could be leveraged to develop a compact, battery‐powered monitor capable of being mounted externally on an oxygen mask, which could accurately detect exhaled gases without additional equipment or sampling lines.

To test this hypothesis, we developed a prototype device utilising a modern MEMS humidity sensor, a microcontroller and a light‐emitting diode (LED) to provide visual breath‐by‐breath indications. The system was designed for seamless integration into existing clinical workflows, intentionally avoiding adding set‐up burden for staff. This pilot study evaluated the feasibility and reliability of the humidity‐based sensor in healthy volunteers to establish a performance baseline prior to patient‐based studies. We hypothesised that this technology could address the monitoring gap by providing a practical, continuous and at‐a‐glance assurance during patient transfer.

## Methods

Ethical approval was granted by the local Human Ethics Committee of the Uniting Care Wesley Hospital (23/10/2020), and written informed consent was obtained from all participants. Fifty adult volunteers were recruited from multiple hospital departments in Brisbane, Australia. Participants included nurses, technicians and anaesthetists with clinical experience in post‐anaesthesia care, surgical wards and emergency settings. These participants were all experienced in using respiratory monitoring equipment. Participants were excluded if they were not experienced in respiratory monitoring or had known respiratory pathology. All participants were over 18 years of age, and no participant had an active respiratory illness at the time of testing.

**Figure 1:**
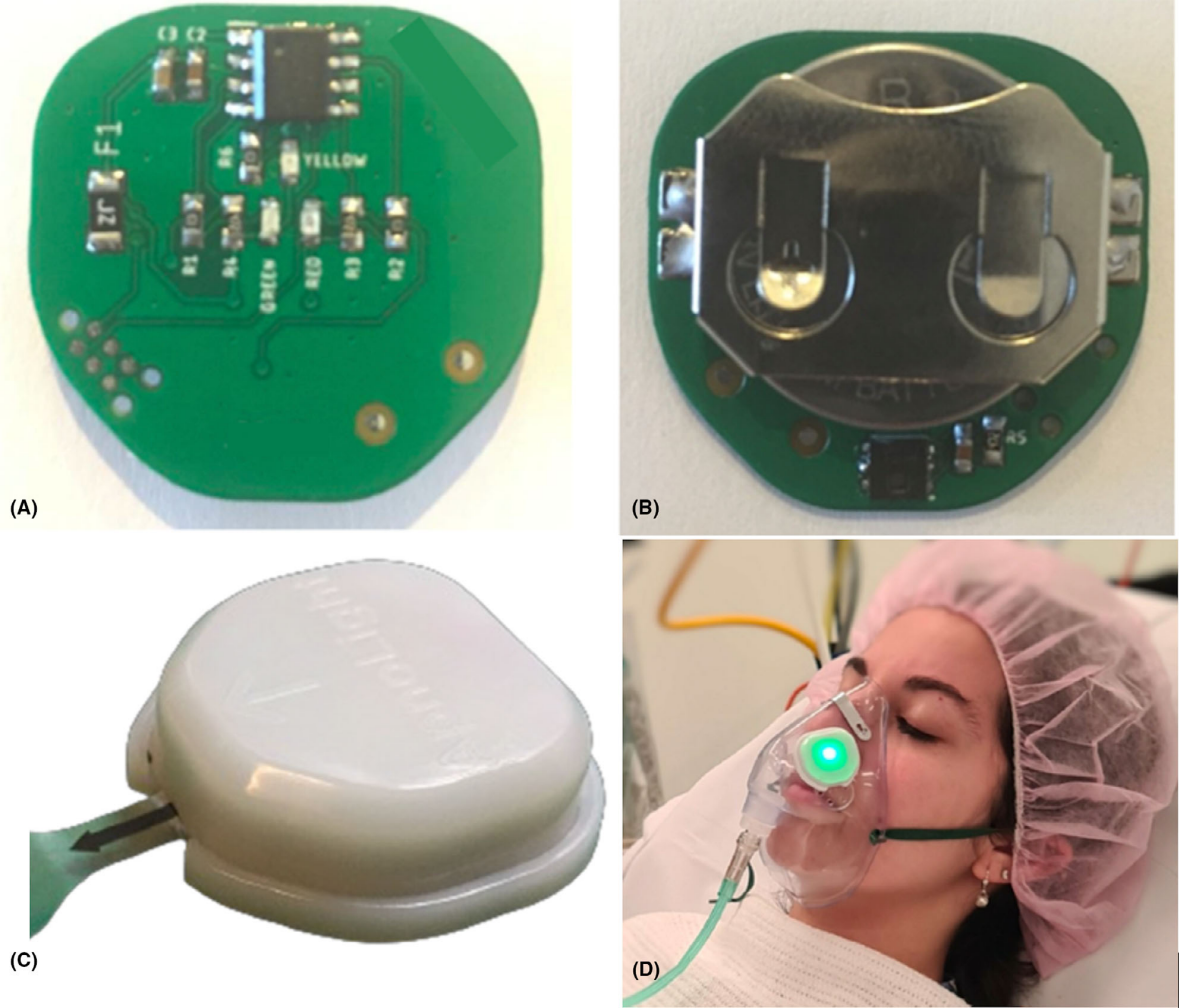
(A) Internal printed circuit board layout highlighting the microcontroller and light indicators. (B) Opposing side of the printed circuit board featuring the 3 V coin‐cell battery and the 3 × 3 mm MEMS humidity sensor. (C) Assembled device with battery isolation tab. (D) Device attached to an oxygen mask indicating a breath with a green light flash.

The experimental device utilised an AHT21 (ASAIR, Aosong Electronics, China) integrated digital humidity sensor mounted on a custom‐printed circuit board (PCB). The system was controlled by an ultra‐low‐power microcontroller and housed in a lightweight, 3D‐printed plastic enclosure designed to adhere onto the external surface of standard oxygen masks (Figure 1). The assembled device weighed 11 g adding negligible weight or discomfort to the oxygen mask and had a total cost of less than $2.00 AUD.

The microcontroller was programmed with a proprietary slope detection algorithm to identify the respiratory cycle in real time. Exhalation was characterised by a rapid increase in relative humidity occurring over several hundred milliseconds, while inhalation was identified by a corresponding decrease as moisture evaporated from the sensor surface. When the rate of change exceeded a predefined threshold, the microcontroller triggered a green LED flash to provide a visual breath‐by‐breath indicator.

The sensing strategy and values were finalised following extensive bench testing. Initial prototyping compared the signal‐to‐noise ratios of temperature and humidity transients using the dual channel inputs of the AHT21. While humidity values from directly over the lateral ventilation ports of the mask fluctuate significantly (range from approximately 50% to 95%), temperature variations were negligible (0.1–0.2°C) and were frequently affected by ambient thermal noise and room currents. This confirmed that humidity transients provided a much more robust and reliable signal for breath detection from an external position on the mask.

For this study, a new device was used for each participant. Devices were initialised by removing a battery isolation tab and required no external calibration, power supply or secondary monitoring equipment, facilitating a seamless integration into workflow.

Participants were fitted with an elongated, medium‐concentration oxygen face mask (Parker Healthcare, Melbourne, Australia). The experimental MEMS‐based device was affixed to the external surface of the mask using medical grade adhesive, positioned to directly overlap the lateral ventilation ports.

To provide a reference standard for comparison, a CO_2_ gas sampling line (Parker Healthcare, Melbourne, Australia) was connected from a GE Healthcare capnography monitor (GE Medical Systems Information Technologies Inc, WI, USA) to the ventilation port on the contralateral side of the mask. Oxygen flow was standardised at 4 l.min^−1^ for all participants.

Correct mask fit was confirmed by the principal investigator to ensure an adequate seal and minimise leakage, which could potentially degrade both the humidity transients and the capnography waveform. In instances where participants possessed facial features which challenged a standard mask seal, such as a narrow facial profile, the fit was documented to allow for sub‐analysis of potential missed breaths during data evaluation. Participants remained supine throughout the recording period to replicate the immediate postoperative state during transfer and early recovery.

To evaluate the device under conditions of clinical observation, participants were assessed in pairs using a reciprocal observer‐subject model. Each participant was briefed on the function of the device and instructed on its application to their individual, single‐use oxygen mask. During each session, one participant assumed a supine position on a hospital bed and was fitted with the prepared oxygen mask. A 2‐minute acclimatisation period was provided to allow the participant to reach a steady‐state natural breathing pattern.

The second participant acted as the observer, tasked with recording the number of green LED flashes emitted by the device over 60 seconds. Simultaneously, the principal investigator, who was blinded to the observer's tally, recorded the respiratory rate and waveform count provided by the capnography monitor. This dual observation strategy ensured that the humidity‐based breath count was captured independently of the reference standard.

Following the initial recording, participants swapped roles and the protocol was repeated so that one respiratory rate observation was recorded for each participant. To ensure methodological consistency, the same principal investigator oversaw all sessions and capnography data collection throughout the study. This procedure yielded a total of 50 paired data points for comparative analysis.

Data were analysed using Microsoft Excel (Version 16.0, Microsoft Corp., WA, USA). Respiratory rate measurements were described using mean, standard deviation (SD) and range. Agreement between the MEMS humidity sensor and reference capnography was evaluated using Bland–Altman analysis to determine mean bias and 95% limits of agreement. Clinically acceptable agreement was defined as per previous studies as a bias and limit of agreement within 3 breaths.min^−1^ [8].

The linear association was assessed using the Pearson correlation coefficient (r). Breath‐by‐breath accuracy was defined as the percentage of 60‐second intervals where the humidity‐derived count was within one breath of the capnography waveform. Data were inspected for outliers, with a focus on the impact of documented clinical artefacts (mask fit) on signal integrity. A p‐value of < 0.05 was considered statistically significant.

## Results

A total of 50 healthcare professionals were recruited to participate in the study, including nurses, clinical technicians and anaesthetists. The cohort represented a wide range of ages (22–66 years) and body habitus, with BMI ranging from 16 to 37 kg.m^−2^. The study participants were predominantly women (88%). All participants successfully completed the monitoring protocol without adverse events or deviations. Participant characteristics and baseline respiratory data are summarised in Table 1.

**Table 1:** Participants' characteristics. Values are presented as mean (SD) or number (%).

	*n* = 50
Age, years	42 (11)
Sex; n (%)	
Male	12 (6)
Female	88 (44)
BMI, kg.m^−2^	27 (5.25)
Respiratory rate, breaths.min^−1^	13 (5)

Data from all 50 participants were included in the final analysis. Across the cohort, the capnography monitor recorded a total of 662 breaths, while the MEMS‐based device recorded 654 breaths. This yielded an overall breath‐by‐breath detection accuracy of 98.8%. Eight false‐negative events (missed breaths) were recorded by the device. Notably, no false‐positive detections occurred. Measured respiratory rates ranged from 8 to 28 breaths.min^−1^, with a standard deviation of 0.68 breaths.min^−1^ across the paired measurements.

Bland–Altman analysis demonstrated strong agreement between the two modalities. The mean bias was 0.16 breaths.min^−1^, with 95% limit of agreement from −1.17 to +1.49 breaths.min^−1^. These limits fell within the predefined clinical threshold of ±3 breaths.min^−1^. Pearson correlation analysis confirmed a strong linear relationship between the humidity‐derived respiratory rate and capnography (r = 0.98, p < 0.001).

Forty‐seven participants out of 50 (94%) exhibited perfect breath‐by‐breath agreement. In the remaining three participants, individual breaths were missed by the device, appearing as outlier data points. In these specific cases, the principal investigator documented a suboptimal mask seal due to facial anatomy with substantial gaps observed around the nose and chin. This interface failure likely allowed exhaled gas to bypass the lateral ventilation ports, reducing the moisture volume reaching the MEMS sensor.

## Discussion

In this pilot study, we evaluated a respiratory monitoring prototype incorporating a MEMS humidity sensor. Compared with capnography, the device demonstrated breath‐by‐breath accuracy of 98.8% with minimal mean bias (0.16 breaths.min^−1^). The 95% limits of agreement (−1.17 to +1.49 breaths.min^−1^) were within the predefined clinically acceptable range of ±3 breaths.min^−1^. These results indicate that measurement of humidity transients in exhaled gas can provide breath detection comparable with capnography in spontaneously breathing adults, supporting further evaluation of this sensing approach when integrated with standard oxygen face masks.

The strong correlation observed is consistent with the close relationship between moisture and CO_2_ in exhaled breath [11]. Earlier humidity‐based systems were limited by sensor sensitivity and response time, often requiring modified delivery systems, whereas contemporary MEMS sensors demonstrate faster response characteristics [12, 13]. This allowed detection of early exhalation moisture changes despite 4 l.min^−1^ supplemental oxygen. Signal contrast arises from the difference between humid exhaled gas and relatively dry medical oxygen and ambient air, in contrast to the dilution effects encountered with side‐stream capnography [6].

This approach may be relevant to the monitoring gap during patient transfer highlighted in the NAP7 report [1]. In contrast to many existing technologies, this device requires no set‐up beyond applying it to a standard oxygen mask, no calibration and no ongoing maintenance. It also does not require additional sampling lines, external monitors or power supply. Because it detects exhaled humidity rather than CO_2_, its performance is not degraded by gas dilution within oxygen masks, which commonly affects side‐stream capnography in spontaneously breathing patients [6]. Unlike accelerometer or impedance‐based systems, it is not dependent on chest wall motion and is therefore less susceptible to artefact during patient transfers. These characteristics allow the device to integrate into current clinical workflows with minimal behavioural change, while its very low manufacturing cost may facilitate wider adoption compared with more complex wearable or monitor‐based systems.

Complete agreement was observed in 47 of 50 participants. Missed detections in three cases were associated with suboptimal mask fit and inadequate routing of exhaled gas across the sensor, indicating a limitation of the current design. Future work should examine enclosure geometry and sensor positioning, including sampling from multiple airflow pathways and exploring options for numerical respiratory rate display.

Several limitations should be noted. As a volunteer study, results may not extrapolate to postoperative patients with altered respiratory patterns or low tidal volumes. Performance in high ambient humidity or during aerosol therapy was not assessed, and the effect of artefacts, such as coughing or speech, remains unknown. Interpretation of visual indicators by clinical staff also requires evaluation. Finally, the study participants were 88% female; therefore, we did not assess the accuracy of the device on a broad population of men, particularly where facial hair may disrupt oxygen mask fit.

This pilot study supports MEMS humidity sensing as an accurate and practical, low‐cost approach to respiratory monitoring which fits with current clinical workflows during patient transfer. However, further evaluation in postoperative patients and high‐risk populations is warranted to determine its impact on early detection of respiratory compromise.
